# Comparation of differences in the performance of corporate social responsibility between Chinese and American pharmaceutical enterprises—based on corporate social responsibility report

**DOI:** 10.3389/fphar.2023.1116466

**Published:** 2023-05-22

**Authors:** HongHao Xu, Meng Zhang, ShuZhen Chu

**Affiliations:** School of International Pharmaceutical Business, China Pharmaceutical University, Nanjing, China

**Keywords:** pharmaceutical company, semantic network analysis, CSR, text mining, information disclosure

## Abstract

**Objective:** We compared Chinese and American pharmaceutical companies’ corporate social responsibility (CSR) reports to determine their differences and to analyze the possible reasons for them.

**Methods:** We took as a model the top 500 pharmaceutical companies from Torreya’s (a global investment bank) list of the 1,000 most valuable pharmaceutical companies in the world. We then collected the 2020 corporate social responsibility reports of 97 Chinese and 94 American pharmaceutical companies. These reports were analyzed using software such as ROST Content Mining 6.0 and Gephi 0.92.

**Results:** We formed a high-frequency word list, a semantic network diagram, and a high-frequency word centrality scale for the Chinese and American pharmaceutical corporate social responsibility reports. The Chinese pharmaceutical companies’ corporate social responsibility reports formed a layout of “double centers and double themes,” and the text paid more attention to the disclosure of environmental protection information. The American pharmaceutical companies formed a report presentation form of “three centers and two themes,” focusing on corporate social responsibility information disclosures from the perspective of humanistic care.

**Discussion:** The differences in between Chinese and American pharmaceutical companies’ corporate social responsibility reports may be due to different corporate development strategies, regulatory requirements, social demands, and the concept of “corporate citizenship.” This study makes recommendations for Chinese pharmaceutical companies to better fulfill their CSR at three levels: policy-making, company management, and society.

## 1 Introduction

The introduction of the “Health China 2030” plan requires that pharmaceutical companies clearly understand the essential characteristics of drugs as special commodities and to consciously favor these characteristics as the companies balance pursuing profits and fulfilling their corporate social responsibility (CSR). To date, a significant number of pharmaceutical companies have recognized their sacred social responsibility to protect people’s health. In the context of the COVID-19 epidemic, many companies donated money and materials and resumed work and production in advance to actively promote anti-epidemic work. However, many pharmaceutical companies have been exposed to scandals, in particular, the Changchun Changsheng biological vaccine counterfeiting case. In such a unique context, concern has gradually increased about pharmaceutical companies’ disclosure and fulfillment of their social responsibilities.

As a carrier of CSR content research, CSR reports have received the attention of many scholars at home and abroad. In the practice of the CSR report research, scholars have explored and summarized some experiences regarding their research methods. Foreign scholars, such as Campbell et al. (2003), have used text analysis combined with the scores of researchers or evaluation agencies to obtain the CSR content disclosure index to evaluate and analyze CSR reports. Domestic scholars, such as Tang et al. (2006); Li and Rui (2007), have used the index method to evaluate CSR reports. Lu Wen Dai et al. (2021) used the text analysis method to evaluate CSR reports by mining and organizing their contents with the help of Python software (Lv and Qian, 2021). In terms of the research on the content of CSR reports, the studies have mainly focused on the relationship between CSR, on the one hand, and corporate performance and innovation, on the other hand, as well as the analysis of CSR impact factors. For example, Yu et al. (2021) concluded that CSR commitment has a positive effect on a company’s innovation ability; Li and Xiaoqin (2018) concluded that CSR commitment has an inverted U-type relationship with the shareholding ratio of corporate executives; and Zhang et al. (2021) concluded that CSR has a significant, positive effect on corporate performance.

In summary, the current research on domestic companies’ CSR reports has mainly used the index method, and the research has been carried out after quantifying the influencing factors of CSR and corporate characteristics such as social responsibility on corporate performance and corporate innovation, while less research has been conducted on the textual content and characteristics of CSR reports. At the same time, the pharmaceutical industry is related to people’s health, but most of the current CSR report research has been done in highly polluting and high energy consumption industries—such as iron, steel, and the emerging financial industry—and relevant research remains lacking on the pharmaceutical industry. In this paper, by checking the results of the industry classification of listed companies announced by the China Securities Regulatory Commission as well as authoritative websites such as Cninfo Information and Sohu, we found the following results: only 47 (21.96%) of the 215 pharmaceutical manufacturing companies listed on the Shanghai and Shenzhen stock markets in 2020 released CSR reports, 11 (44.00%) of the 25 mainland listed pharmaceutical distribution companies released CSR reports, and only 22 (22.00%) of the top 100 Chinese pharmaceutical companies have released CSR reports. These findings show that the current practice of CSR reporting by Chinese pharmaceutical companies remains in the development stage. In the United States, the practice of CSR reporting began earlier than it did in China, as early as 1905, when United States Steel announced in its annual corporate report the establishment of public health facilities as well as a concern for the children of its employees’ labor, health, and education (Zheng, 1990). American pharmaceutical companies have far-reaching influence globally, and their CSR reporting practices and information disclosure have been at the industry’s forefront.

This study compares Chinese and American pharmaceutical companies’ CSR reports to obtain their differences and analyze the possible reasons for them. Therefore, this paper selects Chinese and American pharmaceutical companies’ CSR reports and uses the text analysis method to conduct a comparative study. The text analysis method is widely used to sort and mine policy documents and reports. By mining the target text, we can extract the relevant information needed for the study as well as summarize and discover the development trend and patterns of the textual data, thereby providing ideas for subsequent qualitative and quantitative research (Jinlong et al., 2012; Zhang et al., 2020; Sun and Zhenyan, 2022; Yafei et al., 2022). At the same time, a semantic network analysis derived from the text analysis method is used to explore quantitatively the correlation between the obtained keywords (Liu, 2014), and a further analysis is made of the differences while focusing on the content of the Chinese and American pharmaceutical companies’ CSR reports.

This study clarifies the characteristics of Chinese and American pharmaceutical companies’ CSR reports by comparing the reports. From the perspective of CSR reports, the study enriches the content of the CSR research and provides suggestions for Chinese pharmaceutical companies to better disclose their CSR.

## 2 Word frequency analysis

### 2.1 Sample acquisition

Torreya is an international authoritative investment bank. Its published data have been adopted by many well-known pharmaceutical companies, such as Jiangsu Hengrui Pharmaceuticals Company, Ltd. For the sake of accuracy in data acquisition, the scope of the selected samples was defined as the top 500 pharmaceutical companies from Torreya’s 2020 list of the world’s 1,000 most valuable pharmaceutical companies (released in 2021). According to the countries or regions to which the companies belong, they were divided into China (including Hong Kong, Macao, and Taiwan; *n* = 117) and the United States (*n* = 127). Companies were excluded on the following bases: 1) the company was not listed on any international stock exchange; 2) the company had no relevant information, such as CSR reports; or the company provided only a CSR overview (China, *n* = 20; United States, *n* = 25). Ultimately, we obtained 97 pharmaceutical companies from China and 94 from the United States (see Table 1; figures in brackets are the total number of pharmaceutical companies in the country in the ranking section).

**TABLE 1:** Number of Chinese and American pharmaceutical enterprises included in the statistics.

Country rank	Chinese company	American company
1–100	14 (17)	19 (28)
101–200	23 (26)	18 (23)
201–300	24 (26)	18 (26)
301–400	20 (25)	21 (29)
401–500	16 (23)	18 (21)
Total	97 (117)	94 (127)

### 2.2 High-frequency word processing

For pharmaceutical companies in China (including Hong Kong, Macao, and Taiwan), this article selected SINA Finance and Economy to search the 2020 CSR and annual reports of the selected Chinese companies. The selected companies were listed on Chinese, Hong Kong, or American stock markets, where their CSR reports were filed. These files were collected and integrated into a txt file called “csr_china_text.” Textual processing was conducted on the file through ROST Content Mining (CM) 6.0 software. First, we performed word segmentation on the textual content, adjusted and updated the word segmentation vocabulary according to the content as required by the research and the results of the initial word segmentation, and performed a second word segmentation. Second, the results of the second word segmentation were used to enumerate the word frequency, and those words with high frequencies but no meaning were excluded. Finally, the above operations were repeated to constantly improve the word segmentation and filter lists. The word frequency was enumerated according to the updated word segmentation and filter lists, and the Excel file “csr_china_word frequency” was output.

For American pharmaceutical companies, this paper selects the 2020 CSR or environmental social and governance (ESG) reports by searching the companies’ official websites. The social responsibility text (hereafter referred to as CSR reports) in these reports was integrated into the txt document “csr_usa_text,” which Python software was used to analyze. In the first pass of the word frequency statistics, the Python word frequency statistics code was established, and numbers, single letters, punctuation marks, etc. were set as filter words. Secondly, the results were used to filter out high-frequency but unrelated phrases, thus updating the filtered word list. Finally, these operations were repeated to improve the word frequency statistical results, and the Excel file “csr_usa_word frequency” was output (see Table 2 for the results).

**TABLE 2:** Word frequency analysis results of Chinese and American pharmaceutical companies’ social responsibility texts in 2020 (Top 20).

	Chinese company		American company	
Rank	High-frequency	Word frequency	High-frequency	Word frequency
1	Company	3,224	Employees	4,032
2	Emission	2,917	Patients	3,215
3	Environmental	2,026	Health	2,769
4	Standard	1,361	Program	2,351
5	Poverty Alleviation	1,234	Products	1,977
6	Employees	1,225	Report	1,857
7	Management	1,164	Company	1,689
8	Pollution	1,123	Business	1,529
9	Society	1,015	Safety	1,464
10	Waste Gas	999	Support	1,337
11	Responsibility	995	Management	1,327
12	Sewage	944	Compliance	1,294
13	Company	938	Development	1,059
14	Health	916	Corporate	982
15	Environmental Protection	862	Data	963
16	Pharmacy	852	Clinical	953
17	Protection	843	Quality	879
18	Development	827	Environmental	832
19	Monitor	788	Global	799
20	Security	703	Communities	758

### 2.3 Word frequency characteristics

#### 2.3.1 Chinese pharmaceutical companies

In the 2020 CSR reports of Chinese pharmaceutical companies, “company,” “emission,” and “environment” were the three most frequently occurring words, with frequencies of 3,224, 2,917, and 2,026, respectively. Two of the three words are related to the theme of environmental protection. To achieve long-term, sustainable company development, Chinese pharmaceutical companies attach great importance to environmental protection and hope to actively fulfill their social responsibilities through environmental protection information disclosures in the production process. The words “employee” (*n* = 1,225), “management” (*n* = 1,164), “society” (*n* = 1,015), and “responsibility” (*n* = 995), among others, also ranked highly in the word frequency list, which indicates that Chinese pharmaceutical companies pay full attention to the relationship between companies and employees in the development process and actively fulfill their corporate social responsibilities. The word “poverty alleviation” (*n* = 1,234) appeared among the high-frequency words in fifth place. This result indicates that Chinese pharmaceutical companies attach importance to external stakeholders, actively respond to the national call for poverty alleviation in the process of fulfilling their social responsibilities, and create employment opportunities for poor people and the regions in which they live. The high-frequency words shown in Table 4 reveal that when Chinese pharmaceutical companies perform their social responsibilities, the themes they present can be summarized as “development and environment” and “company and employees.”

#### 2.3.2 American pharmaceutical companies

The top three high-frequency words for the American pharmaceutical companies were “employees” (*n* = 4,032), “patients” (*n* = 3,215), and “health” (*n* = 2,769). The first two indicate that American pharmaceutical companies attach great importance to the care and attention of employees and patients in the developmental process. This result is also closely related to the mainstream developmental concept of American pharmaceutical companies, that is, the concept of corporate citizenship from the stakeholder perspective. American pharmaceutical companies pay more attention than Chinese ones do to humanistic care in the process of writing their CSR reports and think about company development planning from a human perspective. Among the high-frequency words, words such as “products” (*n* = 1,977), “safety” (*n* = 1,464), “support” (*n* = 1,337), and “communities” (*n* = 758) also demonstrate that American pharmaceutical companies participate in community activities as corporate citizens in the developmental process, emphasizing key indicators such as drug safety and their concern for supporting companies upstream and downstream in the supply chain. In terms of corporate governance, American pharmaceutical companies emphasize “program” (*n* = 2,351), “compliance” (*n* = 1,294), and “management” (*n* = 1,257), indicating a more structured and standardized management process through project management. Meanwhile, in the governance process, they emphasize legal compliance and attach importance to communication with government stakeholders.

## 3 Semantic network analysis

### 3.1 Co-word matrix processing

As a statistical calculation method, a co-word matrix can clearly show the relevance and logic of the textual data and provide a data framework for a semantic network analysis. In this paper, the two Excel word frequency files were imported into ROST CM 6.0 as a high-frequency word list, and the two txt documents were loaded together as a basic analytical library to generate a common word matrix of high-frequency words in the two countries’ pharmaceutical countries’ CSR reports, as shown in Tables 3, 4.

**TABLE 3:** Results of co-word matrix included in the text of Chinese pharmaceutical enterprise social responsibility report (part).

Words	Health	Security	Pharmacy	Environmental	…	Sewage	Emission	Waste gas
Health	—	191	44	102	…	22	30	21
Security	191	—	37	125	…	15	23	16
Pharmacy	44	37	—	101	…	62	146	62
Environmental	102	125	101	—	…	43	106	67
…	…	…	…	…	…	…	…	…
Sewage	22	15	62	43	…	—	190	83
Emission	30	23	146	106	…	190	—	251
Waste Gas	21	16	62	67	…	83	251	—

**TABLE 4:** Results of co-word matrix included in the text of American pharmaceutical enterprise social responsibility report (part).

Words	Communities	Report	Compliance	Corporate	…	Development	Program	Data
Communities	—	89	79	88	…	95	123	212
Report	89	—	126	221	…	103	117	139
Compliance	79	126	—	184	…	96	208	95
Corporate	88	221	184	—	…	92	157	77
…	…	…	…	…	…	…	…	…
Development	95	103	96	92	…	—	197	99
Program	123	117	208	157	…	197	—	89
Data	212	139	95	77	…	99	89	—

### 3.2 Semantic network processing

**FIGURE 1:**
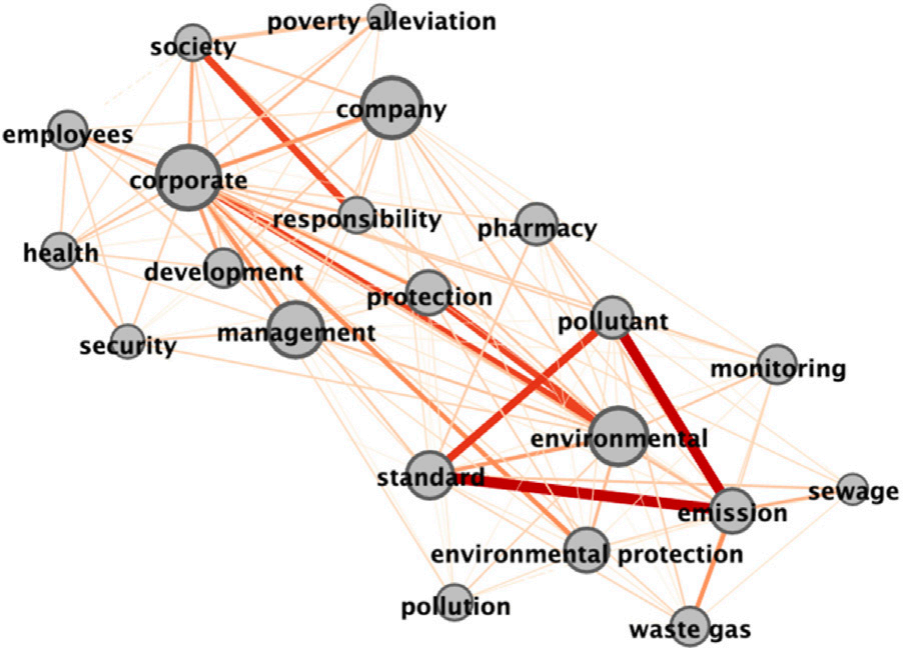
Semantic network diagram incorporated into the social responsibility text of Chinese Pharmaceutical Companies.

**FIGURE 2:**
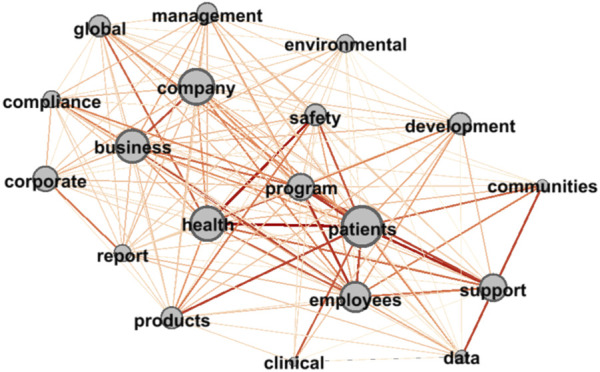
Semantic network diagram incorporated into the social responsibility text of American Pharmaceutical Companies.

Gephi software is used to visualize complex network analyses (Jun et al., 2014). Users can input a prepared common word matrix table into the software and calculate the weights of nodes and edges. When two points of tag words have higher degrees of association, the connections are thicker, the node centrality is higher, and the label is larger (Yuxin et al., 2009; Shenhao and Ronghuan, 2020). In this paper, Gephi 0.92 was used to input the common word matrix table into the software and to output the semantic network diagram of the two countries’ pharmaceutical companies’ CSR reports (see Figures 1, 2).

To further clarify the word frequency characteristics of high-frequency words and to describe the content characteristics of the two countries’ pharmaceutical companies’ CSR reports, this paper uses the quantitative method to quantify each high-frequency word appearing in the semantic network diagram by using ROST CM 6.0 and calculates its point centrality (PC) and intermediary centrality (IC). PC refers to the number of other nodes directly related to a node. Nodes that are connected to more nodes have higher PCs. IC reflects a node’s control ability as a bridge. Nodes with high ICs are located on the shortest paths of many nodes as intermediary nodes. The centrality analysis of semantic network nodes is arranged according to the order of occurrence of each word in the high-frequency word list, and the results are shown in Table 5.

**TABLE 5:** Analysis results of node centrality of semantic network included in Chinese and American pharmaceutical corporate social responsibility texts.

	Chinese company				American company			
Rank	Label	PC	IC		Label	PC	IC	
1	Company	20	23.07143		Employees	18	2.00473	
2	Emission	13	1.66746		Patients	19	3.81517	
3	Environmental	18	11.31191		Health	18	1.91081	
4	Standard	14	3.48412		Program	17	3.43523	
5	Poverty alleviation	6	1.32251		Products	17	1.79524	
6	Employees	11	1.45277		Report	17	0.59371	
7	Management	17	9.23571		Company	18	1.93211	
8	Pollutant	11	0.63888		Business	18	1.93211	
9	Society	10	1.81111		Safety	18	2.35155	
10	Responsibility	10	2.15178		Support	17	0.73251	
11	Sewage	8	0		Management	17	0.73251	
12	Corporate	19	16.26191		Compliance	18	3.36836	
13	Health	10	1		Development	17	1.58631	
14	Environmental protection	13	1.66746		Corporate	15	0	
15	Pharmacy	12	2.11904		Data	16	2.31349	
16	Protection	13	3.79285		Clinical	7	0.33250	
17	Development	11	1.31111		Environmental	17	0.55817	
18	Monitoring	11	0.49285		Global	17	1.03671	
19	Security	9	0.62063		Communities	9	0.08341	

### 3.3 Semantic network characteristics

Through these operations, we obtained the semantic network features of the Chinese and American pharmaceutical company’s CSR reports.

#### 3.3.1 Chinese pharmaceutical companies

Figure 1 shows that the Chinese pharmaceutical companies’ CSR reports presented a distribution of “double centers and double themes,” i.e., they centered on the two keywords “company” and “environmental.” Meanwhile, the remainder of the words was connected around the themes of “environmental protection” and “company management.” Under the latter theme, the keywords “development,” “employees,” “society,” and “management” were closely related. This result indicates that the companies’ CSR reports emphasized that the companies’ development could not be separated from the support of society, the company’s employees, and its management. Figure 1 shows that the connection was thicker between two high-frequency words centered on “environment”; that is, the connection between the high-frequency words in this area was closer than was that of “company.” At the same time, the two centers were connected via “protection” as an intermediary word, forming the theme of “corporate environmental protection.” This finding confirms the assertion that “environmental protection” was the core theme of the CSR reports, as proposed in the word frequency analysis. Among those words, “pollutant” and others indicate that the main pollutant types produced by pharmaceutical companies during their production activities are “waste gas,” “sewage,” etc. The words “standard,” “emission,” and “monitoring” indicate that when companies implement their environmental protection responsibilities, they mainly focus on emission standards and monitoring.

Table 5 shows the prominent intermediary centrality of “company” and “corporate,” indicating that the Chinese pharmaceutical companies’ CSR reports have much content connected by these terms. “Environment” followed immediately, which shows that the companies emphasize the environment as the core of company development in their CSR reports, and the emphasis on environmental protection is the reports’ main content. From the perspective of point degree centrality, there is a certain correlation between the companies’ high-frequency words and the degree of point degree centrality. That is, the words ranked at the top of the high-frequency word list had higher degrees of point degree centrality. “Corporate” was contrary to this trend, and it may be that in the Chinese context, “corporate” and “company” have similar meanings when the legal attributes of the two words require no definition.

#### 3.3.2 American pharmaceutical companies

Overall (See Figure 2), the semantic network diagram of the American pharmaceutical companies’ CSR reports was centered on “safety,” “health,” and “program” and was divided into two parts: the word group of “company” and “business” and that of “patients” and “employees.” The two word groups can be classified as “corporate management” and “humanistic care.” Thus, the American pharmaceutical CSR reports formed a pattern of “three centers and two themes.” In the American pharmaceutical companies’ CSR reports, the three high-frequency central words were more related to “patients” than they were to “company,” which indicates that American pharmaceutical companies give priority to “patients” in their production and operation activities. In the theme group of “company management,” the American pharmaceutical companies mainly emphasized “compliance,” that is, compliance with laws and regulations, as well as giving attention to “environmental” and “management.” In the theme group of “humanistic care,” words such as “people,” “patients,” and “employees” were emphasized. In this word group, the words were more highly correlated. “Support,” “communities,” “products,” and other words demonstrate the role American companies play as “corporate citizens” in the business process mentioned above. In the theme group of “humanistic care,” most of the American pharmaceutical companies’ CSR reports mentioned words related to clinical drug trials such as “clinical” and “data.” This finding indicates that their understanding of clinical drug trials has risen from the perspective of drug safety to the perspective of care for patients and other drug demanders.

From the perspective of the quantitative data (see Table 5), the largest IC was “patients,” which indicates that it had a high control position in the American pharmaceutical companies’ CSR reports. That is, the American pharmaceutical company’s CSR reports focused on “patients.” The subsequent words “program” and “support” once again proved that structured management and supply chain support were keys to the development of the American pharmaceutical companies and highlighted the companies’ role as “corporate citizens” when engaging in business activities in the context of stakeholder theory. The IC of the word “data” also ranked highly, indicating that American pharmaceutical companies pay attention to the use of data when writing their CSR reports. From the perspective of point degree and centrality, the point degree and centrality of the high-frequency words of the American pharmaceutical companies were not much different, and only two words were less than 10. This finding indicates that the American pharmaceutical companies’ CSR reports involved more comprehensive points in their contents and the reports were large in volume. “Patients” and “employees” ranked in the top two, which illustrates that the theme of “humanistic care” occupied a slightly higher proportion than did the other contents in the CSR reports and were in a dominant position.

## 4 Results

Through textual and semantic network analyses, 97 Chinese and 94 American pharmaceutical companies were screened, sorted, and ultimately included in this paper. These companies’ 2020 CSR reports were analyzed. The findings indicate that the Chinese pharmaceutical companies’ CSR reports engaged in much discussion around two centers: “environmental protection” and “corporate management,” especially the former. While the American pharmaceutical companies’ CSR reports included “humanistic care and corporate management” as the theme and “corporate citizenship” as the operating role, forming a “three centers and two themes” model centered on “safety,” “health,” and “program.”(See Table 6).

**TABLE 6:** Comparison of included Chinese and American pharmaceutical corporate social responsibility reports.

	Chinese company	American company
The highest word frequency	“Company” (3,224)	“Employees” (4,032)
Words frequency with more than 2,000 times	3	4
Words frequency with more than 1,000 times	9	13
Word with the highest point centrality	“Company” (20)	“Patients” (19)
Word with the highest intermediary centrality	“Company” (23.07143)	“Patients” (3.81517)
Theme mode	Double center, double theme	Three centers and two themes
Feature theme	Environmental Protection	Humanistic Care
Feature words	“Poverty Alleviation” “Pollutant”	“Patients” “Clinical”

From the perspective of the word frequency results, the number of words related to environmental protection in the high-frequency vocabulary of the Chinese pharmaceutical companies was larger than was that of the American pharmaceutical companies. The Chinese pharmaceutical companies had high-frequency words such as “emission” and “waste gas,” indicating that Chinese pharmaceutical companies should pay more attention to pollutant emissions and regulatory treatment in their daily production and operations than did the American pharmaceutical companies. At the same time, in the context of poverty alleviation, Chinese pharmaceutical companies incorporated national policies into the company action guidelines and proposed poverty alleviation as a concrete action to fulfill their corporate social responsibilities. The American pharmaceutical companies had high-frequency words such as “patients” and “communities,” which were not found in the high-frequency words of the Chinese pharmaceutical companies. This finding indicates that the American pharmaceutical companies’ CSR reports identified more diversified concerned groups. The American pharmaceutical companies’ CSR reports also highlighted on drug quality and clinical trial data. According to the content of the high-frequency vocabulary, although fewer American pharmaceutical companies were included in this article than were Chinese pharmaceutical companies, the difference in keyword frequency was not very large, perhaps due to the following reasons: 1) Chinese and English have different written expression environments. 2) The CSR reports of the American pharmaceutical companies were larger than were those of the Chinese pharmaceutical companies.

The results shown in each high-frequency word centrality table indicate that the Chinese pharmaceutical companies differed greatly in the degree of centrality of high-frequency words, while the American pharmaceutical companies showed a convergence phenomenon under the same indicators. The Chinese pharmaceutical companies had themes such as “company management” and “environmental protection,” emphasized the harmony and unity of the company and the environment, and pursued the company’s long-term sustainable development. The theme was relatively simple. The American pharmaceutical companies, on the other hand, focused on the words “company,” “patients,” and “employees” in the discussion of social responsibility and emphasized the balanced development of shareholders’ and other stakeholders’ interests. The aspects involved were more diverse than were those of the Chinese pharmaceutical companies. Under the intermediary centrality index, the Chinese pharmaceutical companies had the phenomenon of “one super, many giants,” such as “company” (23.07143), “corporate” (16.26191), “environmental” (11.31191), etc., and their intermediary centrality was much higher than was that of the other high-frequency words. The highest intermediary centrality of the American pharmaceutical companies was close to 4, which was lower than that of the Chinese pharmaceutical companies under the same index.

## 5 Discussion

### 5.1 Reasons for the differences in Chinese and American pharmaceutical companies’ CSR reports

This research suggests the following possible reasons for the differences in the performance of social responsibilities between Chinese and American pharmaceutical companies.

#### 5.1.1 Company development strategies

The existing research proposes a certain relationship between corporate development strategy and CSR report disclosures. A company’s scale affects its development strategy. According to stakeholder theory, companies face different stakeholder groups at different developmental stages. As the company scale continually develops, the company finds it easier to face stakeholder supervision and review. For example, Branco and Rodrigues (2008) proposed that companies with larger scales have greater public visibility, so they find it easier to disclose CSR information due to public supervision. When a company is faced with institutional pressure, its immediate response also has a certain impact on its development strategy, leading to a preference in its CSR report focus. For example, Xiaoyue et al. (2022) suggested that in the face of an appeal conflict within the same stakeholder, such as that between a government’s environmental protection and financial departments, the contents of the CSR report show a preference for one party.

#### 5.1.2 Regulatory requirements

American companies have been subject to many legal requirements for social responsibility disclosures in their business activities. For example, the S-K regulation rules promulgated in 1,934 stipulate that listed companies should disclose important information, including debt information, environmental protection efforts and their costs, environmental risks, and legal changes caused by compliance with other laws and regulations. CSR regulations issued in 1971 further legally require CSR in the United States. On the other hand, China’s corresponding CSR disclosure system began in 2006 when the Shenzhen Stock Exchange issued guidance on the social responsibilities of listed companies. Although the guidance stipulates that listed companies should undertake and disclose social responsibility obligations, the provision has not yet been raised to the legal mandatory level. At the same time, Chinese pharmaceutical companies’ CSR reports lack the supervision and management of a third-party review department, so testing the reliability of such reports is challenging.

#### 5.1.3 Social appeal

Due to the drug quality and environmental pollution problems of recent years, Chinese pharmaceutical companies’ CSR reports have consciously inclined toward management and environmental protection. In addition, the strict requirements of government departments for environmental protection data have led to its disclosure and other information in the Chinese pharmaceutical companies’ CSR reports, accounting for the majority of the reports’ contents. American pharmaceutical companies have gradually established more effective drug quality supervision and drug recall systems through many years of operation and management. They also started environmental protection measures earlier than did domestic pharmaceutical companies. Enacting such measures enables pharmaceutical companies to better consider all stakeholders in the CSR reports and to take patients and employees as the core contents of the disclosures.

#### 5.1.4 Corporate citizenship

Corporate citizenship theory, which originated in the West, proposes that companies are rooted in society much like citizens. Companies participate not only in business activities but also in social activities and assume the obligation to contribute to social welfare (Camilleri et al., 2021). This concept is especially relevant to companies who differentiate themselves through responsible and sustainable actions. The American pharmaceutical companies included in this article flaunt their corporate citizenship in their business activities, maintain good interactions with different stakeholders, and accept supervision from all parties.

### 5.2 Suggestions for improving Chinese pharmaceutical companies’ CSR reports

In addition to following the business logic of gaining profits, pharmaceutical companies must clearly realize the essential characteristics of drugs as special commodities, actively undertake CSR in production and operation activities, and timely and effectively disclose CSR information. According to the comparative analysis of the contents of the Chinese and American pharmaceutical CSR reports, we make the following recommendations for CSR disclosures for Chinese pharmaceutical companies.

(1) Policy-makers should speed the improvement of CSR laws and regulations, especially those related to pharmaceutical companies because they are related to people’s health, and guide pharmaceutical companies to actively fulfill their social responsibilities. Specifically, first, policy-makers should speed the establishment of a mandatory disclosure system. Second, policy-makers should improve drug laws and regulations and monitor drug quality at the source. Third, policy-makers should establish incentive policies for CSR information disclosure to encourage companies to disclose information. At the same time, policy-makers should speed the construction of a third-party audit platform for pharmaceutical companies’ social responsibility and improve the reliability of pharmaceutical companies’ CSR reports.
(2) At the company level, domestic pharmaceutical companies should improve their internationalization level and strive to align with the international standards. In the information disclosure of CSR reports, domestic pharmaceutical companies should learn from the practices of their excellent foreign counterparts, that is, by making multi-dimensional and multi-center information disclosures, rather than “compromise” disclosures only for the sake of social hot spots or government requirements. Domestic pharmaceutical companies should make more information disclosures on “humanistic care” from the perspective of consumers (i.e., patients) and employees. In the process of company development, domestic pharmaceutical companies should establish the role of a “corporate citizen” and actively participate in the practice of social responsibility and information disclosure activities.
(3) At the social level, companies should respond to stakeholders’ concerns in the CSR reports. That is, companies should meet the needs not only of internal stakeholders (such as shareholders) but also of suppliers, consumers, communities, and governments. At the same time, through news media, online media, and relevant government departments, society can supervise companies objectively and impartially, expose companies that pollute the environment and produce defective products, and encourage companies that strictly perform their social responsibilities and give back to society.

### 5.3 Limitations

The study of the CSR characteristics of pharmaceutical companies in both countries was limited to their report contents, and the sample size was relatively small. At the same time, the study addresses the Chinese and American pharmaceutical companies’ CSR reports only from the perspective of a textual analysis, lacking further quantitative analysis such as the degree of compliance with regulations or the effectiveness of implementation. In addition, a pharmaceutical company’s CSR disclosures may be affected by the company’s financial status, the company’s influence in the industry, or the company’s reputation.

In subsequent research, researchers can classify the textual content, assign corresponding indicators, and analyze the influencing factors of CSR report disclosure by regression analyses. At the same time, researchers can further study the possible causes of the differences in the disclosure of CSR reports between Chinese and American pharmaceutical companies.

## 6 Conclusion

This study compares and analyzes the CSR reports of pharmaceutical companies in China and the United States from the perspectives of word frequency and network centrality by using text mining and a semantic network analysis. From them, the characteristics are obtained of both countries’ pharmaceutical companies’ CSR reports, and the possible reasons for these characteristics are analyzed. At the same time, the study makes suggestions on how Chinese pharmaceutical companies can better fulfill their CSR and improve their CSR disclosures from three aspects.

In addition to following their business logic of making profits, pharmaceutical companies should clearly understand the essential characteristics of pharmaceuticals as special commodities, actively fulfill their CSR, and disclose CSR information in their production and operation activities. At the same time, domestic pharmaceutical companies should improve their internationalization level and strive to align with international standards. In the disclosure of CSR reports, they should learn from the practices of their excellent foreign counterparts, i.e., by making multi-dimensional and multi-centered information disclosures, rather than simply “compromise” disclosures in response to social hotspots or government requirements. Pharmaceutical companies should disclose more information from the perspective of consumers (i.e., patients), employees, and other aspects of “humanistic care.” In the process of corporate development, pharmaceutical companies should establish themselves in the role of “corporate citizens” and participate in the practice of social responsibility and information disclosure activities in this role. Pharmaceutical companies should pay attention to the needs of various stakeholders and respond to their concerns in CSR reports; i.e., pharmaceutical companies should meet not only the needs of internal stakeholders (e.g., shareholders) but also those of suppliers, consumers, communities, and governments. An independent CSR department should be established within each pharmaceutical company to improve the efficiency of CSR disclosure.

## Data Availability

The raw data supporting the conclusion of this article will be made available by the authors, without undue reservation.
